# Mapping methodologies for economic assessment of digital health technologies: a scoping review protocol

**DOI:** 10.1136/bmjopen-2025-099933

**Published:** 2025-08-01

**Authors:** Ana Rita Santos, Filipa Sampaio, Ana Rita Londral, Julian Perelman

**Affiliations:** 1 Universidade Nova de Lisboa Escola Nacional de Saude Publica, Lisbon, Portugal; 2 Comprehensive Health Research Centre, NOVA University of Lisbon, Lisbon, Portugal; 3 Value for Health CoLAB, Lisbon, Portugal; 4 Department of Public Health and Caring Sciences, Uppsala Universitet, Uppsala, Sweden; 5 Universidade Nova de Lisboa Faculdade de Ciências e Tecnologia, Caparica, Setubal, Portugal

**Keywords:** eHealth, Telemedicine, Artificial Intelligence, Health informatics, Digital Technology, HEALTH ECONOMICS

## Abstract

**Abstract:**

**Introduction:**

Digital health technologies (DHTs) have emerged as transformative tools capable of improving healthcare delivery, reducing costs and enhancing patient care. Despite their potential, the integration of DHTs into healthcare systems is challenged by the lack of clear frameworks for evaluating their cost-effectiveness. Economic evaluations are crucial to guide the adoption and financing of these technologies. However, conventional frameworks often fail to address the unique characteristics and complexities of DHTs fully. This scoping review aims to map the existing frameworks for economic evaluation of DHTs and identify methodological approaches that can inform and guide future assessments.

**Methods and analysis:**

We will conduct a scoping review following the Joanna Briggs Institute methodology and report findings according to the Preferred Reporting Items for Systematic Review and Meta-Analysis Protocols and Preferred Reporting Items for Systematic Reviews and Meta-Analysis for Scoping Reviews guidelines. A systematic search will be conducted in electronic databases (PubMed, Web of Science, Scopus and Business Source Complete) and grey literature sources for studies published since 2015. Included studies must focus on health technology assessment or economic evaluation frameworks tailored to DHTs, addressing economic evaluation methodologies. The data will be extracted using a customised template, and a narrative summary, along with descriptive statistics, will be employed to synthesise the findings.

**Ethics and dissemination:**

Since this review involves publicly available data, ethical approval is not required. The findings will be disseminated through peer-reviewed publications and presentations at relevant conferences. This review aims to provide evidence-based recommendations for integrating DHTs into healthcare systems, influencing policy and guiding future research in health economics.

**STRENGTHS AND LIMITATIONS OF THIS STUDY**

- A multilingual search strategy spanning six languages broadens the scope and diversity of included studies, increasing the comprehensiveness of findings.
- The review employs a robust and transparent methodology grounded in the Joanna Briggs Institute framework and follows established reporting guidelines (the Preferred Reporting Items for Systematic Review and Meta-Analysis Protocols and Preferred Reporting Items for Systematic Reviews and Meta-Analysis for Scoping Reviews), ensuring rigour and reproducibility.
- No quality assessments will be conducted on the included studies, as the objective is to provide a baseline understanding of the state of economic evaluation frameworks in DHTs.

## Introduction

 The discourse on healthcare systems is strongly shaped by the need to maintain economic sustainability. Digital transformation is seen as a promising solution to improve health outcomes and healthcare efficiency.^1^ Digital health (DH) has the potential to address economic challenges by enhancing access to care, streamlining processes, optimising resource use, improving the quality of care and shortening diagnostic delays. It also offers opportunities for more personalised healthcare, benefiting both patients and healthcare systems.^2^,^4^

DH has evolved rapidly and may change how healthcare services are used, allowing individuals to be empowered in monitoring and managing their own care.^4^ According to the WHO, DH is ‘the field of knowledge and practice associated with the development and use of digital technologies to improve health’^5^ and digital health technologies (DHTs) encompass the utilisation of technology for diagnosing, monitoring, treating and preventing illnesses in various forms, such as mobile health (mHealth), electronic health records, wearable or implanted devices with wireless communication capabilities, smartphones equipped with specialised applications (apps), telehealth, telemedicine, artificial intelligence (AI) and robotics.^2 3^

DHTs can enhance health outcomes by fostering greater patient engagement in self-care and caregiving, facilitating better communication and customising services to meet individual requirements.^6^ Yet, despite their potential benefits, DHTs face several challenges, including issues with technical reliability, algorithm transparency, access and usability, workflow and infrastructure adjustments, and security risks in data transmission and storage.^7^ Furthermore, their high development costs present a challenge to health systems worldwide, which struggle with finite resources.^8 9^

In Europe, the pace of digital transformation in healthcare systems is uneven due to several factors: underdeveloped regulatory frameworks, insufficient investment, inadequate infrastructure for data management and a lack of necessary skills among both professionals and users. Moreover, the absence of strong evidence on the true benefits and cost-effectiveness of DHTs complicates decision-making, potentially leading to the adoption of short-lived or low-value technologies.^10^

Prioritising value-based evaluation is essential for the integration of the DHTs into healthcare systems and ensuring the sustainability of the innovation sphere.^11^ For conventional healthcare interventions, achieving this objective has been accomplished through health technology assessment (HTA) to generate scientific evidence regarding the effectiveness, and cost-effectiveness, as well as the organisational, ethical, legal and social implications of health technologies utilisation.^12^ HTA is essential in informing decision-makers of the potential impact of DHTs on both clinical and economic health outcomes.^13^

HTA is typically conducted using established frameworks, which guide the methodologies and evaluation criteria. While these frameworks are valuable for assessing various health technologies, they have limitations in fully addressing the complexities of DHTs. For instance, frameworks like the Evidence Standard Framework of the National Institute for Health and Care Excellence^14^ are often designed for specific socioeconomic or national contexts, reflecting local constraints and preferences, which limit their transferability. Additionally, some frameworks exclude certain types of DHTs or lack sufficient evidence of their practical effectiveness, restricting their broader applicability.^10^

Economic evaluation, a critical component of the HTA process, encounters a similar lack of clear methodological guidance, whether conducted within or independently of the HTA framework. Economic evaluation compares the costs and outcomes of interventions, reflecting the local value of DHTs, facilitating adoption decisions and optimising resource use for global health.^9^ However, the lack of clear guidelines for evaluating DHTs may complicate decision-making, hinder the determination of value for money and lead to suboptimal outcomes. Developing well-defined frameworks is essential to ensure accurate comparisons of cost-effectiveness between healthcare alternatives, enabling more informed decisions in healthcare systems.^9 15 16^

Most economic evaluations concerning DHTs adhere to conventional methodological guidelines for evaluating healthcare technologies, such as drugs and medical devices. Frequently, these evaluations adopt a health service or payer perspective, focus on patient-related health benefits and employ cost-effectiveness analysis with outcomes expressed in quality-adjusted life years. Nonetheless, these methodological premises might not fully capture the unique characteristics of DHTs.^15^

DHTs differ from conventional interventions due to their rapid evolution, user-driven nature, dynamic interactions with users, unique pricing structures and non-health impacts, complicating cost-effectiveness estimation.^15 17^ Due to their iterative development and varied user base, conducting Randomised Controlled Trials (RCTs) is often impractical, leading to reliance on non-randomised studies with inherent methodological concerns.^9 15^ Additionally, the conventional cost-effectiveness approach, which primarily focuses on clinical outcomes, may not adequately capture the broad benefits of DHTs, including non-health-related advantages.^1^ This complexity presents significant challenges for conventional economic evaluation methods, particularly in selecting appropriate comparators, study designs and perspectives, as well as accurately measuring costs and effects.^15^ As such, tailored frameworks are essential to address these challenges and ensure rigorous, relevant assessments of DHTs.

To the best of our knowledge, previous reviews^1 7 10 11 13 18^ have addressed DHTs from an HTA or reimbursement perspective, and none have specifically focused on identifying economic evaluation frameworks that were explicitly developed for or tailored to DHTs. Some of these reviews concentrated on broader HTA domains or implementation barriers, while others examined reimbursement or value assessment without mapping the methodological structure of economic evaluation frameworks for DHTs.

Further research is needed to develop a standardised economic evaluation framework, either independently or within the HTA framework, for DHTs. This would facilitate their integration into healthcare systems and unlock broader economic benefits. By first mapping and characterising existing frameworks that have been explicitly developed or adapted for DHTs, this scoping review aims to lay the foundation for such future developments and guide methodological refinement in this evolving field.

## Objectives

The primary objective of this scoping review is to map existing HTA frameworks that incorporate economic aspects, such as partial or full economic evaluations, and frameworks specifically designed for the economic evaluation of DHTs. This mapping will support a better understanding of their methodological characteristics, strengths and limitations. The findings aim to inform future methodological developments and support the creation of suitable frameworks for the economic evaluation of DHTs.

## Methods

### Study design

We propose to conduct a scoping review to map existing economic evaluation methodologies for DHTs. Scoping reviews are particularly suited to systematically explore the extent, range and nature of existing or emerging literature, especially in fields where evidence is still developing.^19^,^21^ In contrast to systematic reviews, which address specific and narrowly defined research questions, scoping reviews allow for a broader examination of available evidence.^22^ Given that the literature on economic evaluation frameworks for DHTs has not yet been comprehensively reviewed, a scoping review is considered the most appropriate methodological approach for this study.

The conduct of this protocol was guided by the Joanna Briggs Institute (JBI) Evidence Synthesis methodology for scoping reviews^23^ and reported using the Preferred Reporting Items for Systematic Review and Meta-Analysis Protocols (PRISMA-P).^24^ This scoping review will also be conducted with JBI methodology for scoping reviews^23^ and following guidelines outlined in the revised Preferred Reporting Items for Systematic Reviews and Meta-Analysis for Scoping Reviews (PRISMA-ScR)^25^ to ensure transparency and reproducibility of the findings. The PRISMA-ScR checklist will also be employed. This protocol is registered on the Open Science Framework (OSF) (https://osf.io/cejb8) since the PROSPERO database does not accept scoping review protocols. OSF serves as an open-source platform enabling researchers to openly share protocols and data and contribute to the transparency of research objectives.^26^ This study is scheduled for 1 October 2025.

### Research questions

- Which economic evaluation methodologies are included in the frameworks designed to assess DHTs?
- What are the key dimensions (eg, comparator, perspective and time horizon) typically included in existing economic evaluation frameworks?
- How does the choice of economic evaluation methods and cost components in these frameworks vary based on healthcare system characteristics, geographic context and the specific type of DHTs assessed?

### Eligibility criteria

The previously outlined research questions will guide the criteria for selecting studies, adhering to the population, concept and context (PCC) format (table 1). JBI recommends the PCC framework as a guide to construct clear and meaningful objectives and eligibility criteria for conducting a scoping review^23 27^

**Table 1:** PCC framework

Characteristics	Included	Excluded
Population	DHTs	NA
Concept	Economic evaluation frameworks for DHTs HTA frameworks that incorporate economic aspects (specific cost analysis or partial/full economic evaluations) Systematic reviews with economic evaluation frameworks or HTA frameworks	Frameworks that do not include economic aspects, such as cost analysis or economic evaluations (whether full or partial) Studies that fail to propose any methodology involving economic considerations will also be excluded
Context	NA	NA
Type of evidence	Published studies, reports, guidelines and grey literature that include economic aspects for DHTs.	Cost analysis of a specific technology
Language	English, Portuguese, Spanish, French, Swedish and German	Other languages
Date range	2015 to present, focusing on more recent developments in DHTs’ economic aspects	Studies/frameworks published before 2015

DHTs, digital health technologies; HTA, health technology assessment; NA, not applicable; PCC, population, concept and context.

In this study, studies that do not meet the outlined inclusion criteria will be excluded. This includes studies lacking economic components (full or partial economic evaluations) of DHTs, those published in languages other than English, Portuguese, Spanish, French, Swedish and German and those without full-text access. Additionally, other forms of publications such as RCTs, letters, comments, editorials, narrative reviews, study protocols, conference abstracts and papers, posters and presentations will also be excluded.

### Search strategy

A search will be conducted for published literature in the following electronic databases: PubMed, Web of Science, Scopus and Business Source Complete. Studies published with the keywords or Medical Subject Headings (MeSH) terms for three topics (see online supplemental material):

1. (“Digital Health” OR “digital intervention” OR “digital health technology” OR “digital medicine” OR “digital medical devices” OR “digital care” OR “digital therapeutic” OR “eHealth” OR “e-health” OR “mobile health” OR “Mobile Applications” OR “mhealth” OR “m-health” OR “Health Information Systems” OR “telehealth” OR “Telemedicine” OR “telecare” OR “virtual health” OR “Artificial Intelligence” OR “Internet of Things”)

AND

1. (“Costs and Cost Analysis” OR “cost-effectiveness analysis” OR “cost measure” OR “cost comparison” OR “cost minimization analysis” OR “cost analysis” OR “economic analysis” OR “economic evaluation” OR “economic assessment” OR “reimbursement” OR “health technology assessment” OR “budget impact” OR “market access” OR “technology assessment” OR “Technology Assessment, Biomedical” OR “economic impact” OR “value assessment” OR “economic value”)

AND

1. (“Health Policy” OR “guidelines” OR “framework” OR “checklist” OR “recommendation” OR “policy statement” OR “standards” OR “assessment model”)

Each database’s characteristics related to syntax, controlled vocabulary and proximity operators will be considered. The identification of relevant studies will consider materials in English, Portuguese, Spanish, French, Swedish and German published between 2015 and the present. The review is limited to recently published frameworks because of the rapid development of DHTs in the past few years.

The search will be peer-reviewed according to the Peer Review of Electronic Search Strategy checklist to ensure the search is comprehensive and maximises appropriate search terms.^28^

We will carry out a focused search of the grey literature, including publications from international organisations (eg, WHO) and national institutions (eg, Haute Autorité de Santé). Relevant documents will be identified through two main approaches: (1) exploring the websites of regulatory bodies, HTA agencies, government and non-government healthcare organisations, industry groups and consultancies and (2) performing a country-specific Google search informed by the WHO Digital Health Country Profiles report,^29^ which will further refine and enhance the search process. Moreover, a search will be conducted using Google Scholar, limiting the results to the first 200 entries to ensure the inclusion of all relevant studies while maintaining a manageable scope for review. The search terms for the supplementary searches in Google Scholar will consist of combining keywords related to three main concepts: “economic” or “assessment” or “health technology assessment”; “framework” or “guideline”; and “digital health technologies” or “digital medical devices”.

### Study selection

Citations from the literature search will be uploaded to Rayyan (https://www.rayyan.ai/), an AI-assisted systematic literature review tool. Duplicate records will then be identified and removed. The study selection process involves two phases. In the first phase, the studies will be selected by single-blind peer review of the titles and abstracts of the references identified in the bibliographic search. A full-text single-blind review of the studies included in the first phase will be conducted in the second phase. Citations and full-text articles will be screened in duplicate by two reviewers. In the case of disagreement between two reviewers, a consensus will be reached by adding a third reviewer.

### Data extraction

Studies that meet the inclusion criteria will be recorded in an Excel datasheet tailored for this purpose, aligning with the recommendations outlined in the Cochrane Handbook for Systematic Reviews.^30^ The data extraction template will encompass all variables relevant to the research questions and objectives of this scoping review. An iterative process will be undertaken to revise the form as necessary, even during the data extraction phase, to capture any emerging themes not initially identified. The extracted data will include the fields detailed in table 2. Two reviewers will independently extract and code the data. They will then meet to discuss and resolve any discrepancies. If disagreements persist, a third reviewer will be consulted, if necessary, to determine the most appropriate approach to data coding.

**Table 2:** Data charting form

Section	Description
Bibliographic information	
Study ID	
Author, year	
Title of publication	
Type of publication (journal article, book chapter, HTA report, guidance document, etc)	
Language	
Geographic location (country)	
Affiliation (research group/governmental organisation)	
DHTs	
DHTs included	
Healthcare setting (if applicable)	
Economic evaluation	
Type of economic aspects (costs or full economic evaluation)	
Are economic aspects mandatory in DHT evaluations? (yes/no)	
Type of analysis (CEA, CBA, BIA, etc)	
Accepted study designs	
Comparator definition	
Time horizon definition	
Preferred perspective	
Effectiveness measure and source of evidence	
Type of resource use measurement and valuation, and source of evidence	
Uncertainty measurement	

BIA, budget impact analysis; CBA, cost-benefit analysis; CEA, cost-effectiveness analysis; DHTs, digital health technologies.

### Data synthesis

This phase synthesises the findings to explore familiar and unfamiliar aspects of the topic, identifying areas for further investigation, with a narrative summary and numerical summaries to align the results with the review’s objectives and research questions. Dimensions extracted from systematic reviews, which originate from primary studies identified within this systematic search, will be counted only once to mitigate the risk of data duplication and bias. On completing the scoping review, the findings will be presented visually through graphs, diagrams or tables. The results will then be refined and finalised as part of the review process. Finally, the implications of the findings will be discussed concerning the study’s objectives, highlighting potential directions for future research, policy development and practical applications.

### Quality assessment

Peters *et al*^31^ point out that while many scoping reviews encompass a wide array of sources for screening and inclusion, they do not entail statistical pooling, formal risk of bias rating or quality of evidence assessment. Since the objective of this research is to map the existing studies on the economic aspects to evaluate DHTs, this scoping review will not formally evaluate the methodological quality or the risk of bias of the included studies.

## Results

This project assured funding in October 2024. The preliminary scoping search conducted in November 2024 revealed an adequate amount of literature to address the research questions.

## Discussion

DHTs can transform healthcare delivery by enabling remote monitoring, enhancing disease management and offering more tailored treatment options, but their integration into healthcare systems is limited.^1 6^ The opportunity cost of investing in DHTs may be significant, and it is essential to ensure they deliver expected benefits and value for money.^9^ For conventional healthcare interventions, HTA has been instrumental in generating robust scientific evidence on their effectiveness and cost-effectiveness, as well as examining the organisational, ethical, legal and social implications of their adoption. However, the frameworks traditionally employed in the HTA process struggle to address the unique challenges posed by DHTs. These challenges include the rapid pace of technological innovation, the self-learning and adaptive nature of many DHTs, their diversity and novelty, and issues surrounding accessibility and payment.^12 32^

Similarly to the frameworks used in the HTA process, the adaptation and extension of guidelines for designing and conducting standalone economic evaluations tailored to DHTs have been largely overlooked.^16^

The proposed scoping review seeks to identify and analyse frameworks that incorporate economic aspects of DHTs, addressing the existing gap in robust evidence concerning costs and cost-effectiveness measurement methods and guidelines. By mapping available frameworks, this review aims to offer methodological recommendations that can guide future economic studies and support evidence-based decisions on DHTs financing, adoption and scaling up. Importantly, the review also aims to investigate how these frameworks differ across various country contexts, health systems and DHT types. Recognising that methodological soundness alone does not guarantee uptake, the review will consider these contextual and system-level factors as part of the synthesis and interpretation.

## Strengths and limitations

To our knowledge, this is the first scoping review to systematically explore economic evaluation frameworks specifically tailored to DHTs. By focusing on this emerging field, the review provides a state-of-the-art synthesis that will guide researchers, policymakers and developers in selecting appropriate frameworks for assessing the value and feasibility of DHTs. The methodology follows rigorous and transparent approaches, grounded in the JBI framework and adhering to PRISMA-P and PRISMA-ScR guidelines, ensuring comprehensive and reproducible findings. Furthermore, the multilingual search strategy, covering six languages, enhances the inclusiveness and scope of the review.

Despite these strengths, the review may have several limitations. No quality assessments will be conducted on the included studies, as the objective is to provide an initial understanding of the state of economic evaluation frameworks. Moreover, challenges may arise from inconsistencies in terminology across the literature, which could hinder the identification of relevant frameworks.

## Ethics and dissemination

The review seeks to provide actionable, evidence-based recommendations for integrating DHTs into healthcare systems, influencing policy development and informing future research in health economics.

This scoping review examines methodological frameworks—either standalone or embedded within the HTA process—used to evaluate DHTs through cost analyses or economic evaluations. It does not involve collecting primary data or engaging with patients. Since the review relies exclusively on publicly accessible materials, ethical board approval is not required.

The dissemination plan is designed for end-users and incorporates both passive and interactive methods, such as publishing in peer-reviewed journals, presenting at conferences and participating in networking events with stakeholders in DH and health economics.

## Supplementary material

10.1136/bmjopen-2025-099933 online supplemental file 1
