# Supplementary material for: Mapping methodologies for economic assessment of digital health technologies: a scoping review protocol
Source: BMJ Open. 2025 Aug 1;15(7):e099933. doi: 10.1136/bmjopen-2025-099933 (PMC12315032; doi:10.1136/bmjopen-2025-099933)
Supplement: online supplemental file 1 [file bmjopen-15-7-s001.pdf]

SUPPLEMENTARY MATERIAL

APPENDIX 1: SEARCH STRATEGY

PubMed: Search conducted on 29 November, year 2024.

Search	Query	Records Retrieved
#1	("Digital Health"[MeSH Terms] OR "digital intervention"[Title/Abstract] OR "digital health technology"[Title/Abstract] OR "digital medicine"[Title/Abstract] OR "digital medical devices"[Title/Abstract] OR "digital care"[Title/Abstract] OR "digital therapeutic"[Title/Abstract] OR "eHealth"[Title/Abstract] OR "e-health"[Title/Abstract] OR "mobile health"[Title/Abstract] OR "Mobile Applications"[MeSH Terms] OR "mhealth"[Title/Abstract] OR "m-health"[Title/Abstract] OR "telehealth"[Title/Abstract] OR "Telemedicine"[MeSH Terms] OR "Health Information Systems"[MeSH Terms] OR "telecare"[Title/Abstract] OR "virtual health"[Title/Abstract] OR "Artificial Intelligence"[MeSH Terms] OR "Internet of Things"[MeSH Terms])	303.123
#2	("Costs and Cost Analysis"[MeSH Terms] OR "cost-effectiveness analysis"[Title/Abstract] OR "cost measure"[Title/Abstract] OR "cost comparison"[Title/Abstract] OR "cost minimization analysis"[Title/Abstract] OR "cost analysis"[Title/Abstract] OR "economic analysis"[Title/Abstract] OR "economic evaluation"[Title/Abstract] OR "economic assessment"[Title/Abstract] OR "health technology assessment"[Title/Abstract] OR "budget impact"[Title/Abstract] OR "market access"[Title/Abstract] OR "technology assessment"[Title/Abstract] OR "Technology Assessment, Biomedical"[MeSH Terms] OR "reimbursement"[Title/Abstract] OR “economic impact” [Title/Abstract] OR “value assessment”[Title/Abstract] OR “economic value”[Title/Abstract])	341.258
#3	("health policy"[MeSH Terms] OR "guidelines"[Title/Abstract] OR "framework"[Title/Abstract] OR "checklist"[Title/Abstract] OR "recommendation"[Title/Abstract] OR "policy	1.286.089

	statement"[Title/Abstract] OR "standards"[Title/Abstract] OR “assessment model”[Title/Abstract])	
#1 AND #2 AND #3		945
#1 AND #2 AND #3 AND FILTER (2015 TO PRESENT AND ENGLISH, PORTUGUESE, SPANISH, FRENCH, SWEDISH AND GERMAN)		642

Web of Science: Search conducted on 29 November, year 2024.

Search	Query	Records Retrieved
#1	("Digital Health" OR "digital intervention" OR "digital health technology" OR "digital medicine" OR "digital medical devices" OR "digital care" OR "digital therapeutic" OR "eHealth" OR "e-health" OR "mobile health" OR "Mobile Applications" OR "mhealth" OR "m-health" OR "Health Information Systems" OR "telehealth" OR "Telemedicine" OR "telecare" OR "virtual health" OR "Artificial Intelligence" OR "Internet of Things") (Topic)	415.596
#2	("Costs and Cost Analysis" OR "cost-effectiveness analysis" OR "cost measure" OR "cost comparison" OR "cost minimization analysis" OR "cost analysis" OR "economic analysis" OR "economic evaluation" OR "economic assessment" OR "reimbursement" OR "health technology assessment" OR "budget impact" OR "market access" OR "technology assessment" OR "Technology Assessment, Biomedical" OR “economic impact” OR “value assessment” OR “economic value”) (Topic)	209.178
#3	("Health Policy" OR "guidelines" OR "framework" OR "checklist" OR "recommendation" OR "policy statement" OR "standards" OR "assessment model") (Topic)	3.227.878
#1 AND #2 AND #3		849
#1 AND #2 AND #3 AND FILTERS (2015 TO PRESENT AND ENGLISH, PORTUGUESE, SPANISH, FRENCH, SWEDISH AND GERMAN)		730

Scopus: Search conducted on 29 November, year 2024.

Search	Query	Records Retrieved
#1	((TITLE-ABS("Digital Health" OR "digital intervention" OR "digital health technology" OR "digital medicine" OR "digital medical devices" OR "digital care" OR "digital therapeutic" OR "eHealth" OR "e-health" OR "mobile health" OR "Mobile Applications" OR "mhealth" OR "m-health" OR "Health Information Systems" OR "telehealth" OR "Telemedicine" OR "telecare" OR "virtual health" OR "Artificial Intelligence" OR "Internet of Things")) OR (AUTHKEY("Digital Health" OR "digital intervention" OR "digital health technology" OR "digital medicine" OR "digital medical devices" OR "digital care" OR "digital therapeutic" OR "eHealth" OR "e-health" OR "mobile health" OR "Mobile Applications" OR "mhealth" OR "m-health" OR "Health Information Systems" OR "telehealth" OR "Telemedicine" OR "telecare" OR "virtual health" OR "Artificial Intelligence" OR "Internet of Things")))	643.464
#2	((TITLE-ABS("Costs and Cost Analysis" OR "cost-effectiveness analysis" OR "cost measure" OR "cost comparison" OR "cost minimization analysis" OR "cost analysis" OR "economic analysis" OR "economic evaluation" OR "economic assessment" OR "reimbursement" OR "health technology assessment" OR "budget impact" OR "market access" OR "technology assessment" OR "Technology Assessment, Biomedical" OR “economic impact” OR “value assessment” OR “economic value”)) OR (AUTHKEY("Costs and Cost Analysis" OR "cost-effectiveness analysis" OR "cost measure" OR "cost comparison" OR "cost minimization analysis" OR "cost analysis" OR "economic analysis" OR "economic evaluation" OR "economic assessment" OR "reimbursement" OR "health technology assessment" OR "budget impact" OR "market access" OR "technology assessment" OR "Technology Assessment, Biomedical" OR “economic impact” OR “value assessment” OR “economic value”)))	292.495

#3	((TITLE-ABS("Health Policy" OR "guidelines" OR "framework" OR "checklist" OR "recommendation" OR "policy statement" OR "standards" OR "assessment model") OR AUTHKEY("Health Policy" OR "guidelines" OR "framework" OR "checklist" OR "recommendation" OR "policy statement" OR "standards" OR "assessment model")))	7.665.978
#1 AND #2 AND #3		1.551
#1 AND #2 AND #3 AND FILTERS (2015 TO PRESENT AND ENGLISH, PORTUGUESE, SPANISH, FRENCH, SWEDISH AND GERMAN)		1.319

Business Source Complete: Search conducted on 29 November, year 2024.

Search	Query	Records Retrieved
#1	TI ("Digital Health" OR "digital intervention" OR "digital health technology" OR "digital medicine" OR "digital medical devices" OR "digital care" OR "digital therapeutic" OR "eHealth" OR "e-health" OR "mobile health" OR "Mobile Applications" OR "mhealth" OR "m-health" OR "Health Information Systems" OR "telehealth" OR "Telemedicine" OR "telecare" OR "virtual health" OR "Artificial Intelligence" OR "Internet of Things") OR AB ("Digital Health" OR "digital intervention" OR "digital health technology" OR "digital medicine" OR "digital medical devices" OR "digital care" OR "digital therapeutic" OR "eHealth" OR "e-health" OR "mobile health" OR "Mobile Applications" OR "mhealth" OR "m-health" OR "Health Information Systems" OR "telehealth" OR "Telemedicine" OR "telecare" OR "virtual health" OR "Artificial Intelligence" OR "Internet of Things") OR KW ("Digital Health" OR "digital intervention" OR "digital health technology" OR "digital medicine" OR "digital medical devices" OR "digital care" OR "digital therapeutic" OR "eHealth" OR "e-health" OR "mobile health" OR "Mobile Applications" OR "mhealth" OR "m-health" OR "Health Information Systems" OR "telehealth" OR "Telemedicine" OR "telecare" OR "virtual health" OR "Artificial Intelligence" OR "Internet of Things")	99.807
#2	TI (("Costs and Cost Analysis" OR "cost-effectiveness analysis" OR "cost measure" OR "cost comparison" OR "cost minimization analysis" OR "cost analysis" OR "economic analysis" OR "economic evaluation" OR "economic assessment" OR "reimbursement" OR "health technology assessment" OR "budget impact" OR "market access" OR "technology assessment" OR "Technology Assessment, Biomedical" OR “economic impact” OR “value assessment” OR “economic value”)) OR AB (("Costs and Cost Analysis" OR "cost-effectiveness analysis" OR "cost measure" OR "cost comparison" OR "cost minimization analysis" OR "cost analysis" OR "economic analysis" OR "economic evaluation" OR "economic assessment" OR "reimbursement" OR "health	63.761

	technology assessment" OR "budget impact" OR "market access" OR "technology assessment" OR "Technology Assessment, Biomedical" OR “economic impact” OR “value assessment” OR “economic value”)) OR KW (("Costs and Cost Analysis" OR "cost-effectiveness analysis" OR "cost measure" OR "cost comparison" OR "cost minimization analysis" OR "cost analysis" OR "economic analysis" OR "economic evaluation" OR "economic assessment" OR "reimbursement" OR "health technology assessment" OR "budget impact" OR "market access" OR "technology assessment" OR "Technology Assessment, Biomedical" OR “economic impact” OR “value assessment” OR “economic value”))	
#3	TI (("Health Policy" OR "guidelines" OR "framework" OR "checklist" OR "recommendation" OR "policy statement" OR "standards" OR "assessment model")) OR AB (("Health Policy" OR "guidelines" OR "framework" OR "checklist" OR "recommendation" OR "policy statement" OR "standards" OR "assessment model")) OR KW (("Health Policy" OR "guidelines" OR "framework" OR "checklist" OR "recommendation" OR "policy statement" OR "standards" OR "assessment model"))	675.290
#1 AND #2 AND #3		49
#1 AND #2 AND #3 AND FILTERS (2015 TO PRESENT AND ENGLISH, PORTUGUESE, SPANISH, FRENCH, SWEDISH AND GERMAN)		43
