# Supplementary material for: Mapping methodologies for economic assessment of digital health technologies: a scoping review protocol
Source: BMJ Open. 2025 Aug 1;15(7):e099933. doi: 10.1136/bmjopen-2025-099933 (PMC12315032; doi:10.1136/bmjopen-2025-099933)
Supplement: Supplementary file 2 [file bmjopen-2025-099933.reviewer_comments.pdf]

PEER REVIEW HISTORY

BMJ Open publishes all reviews undertaken for accepted manuscripts. Reviewers are asked to complete a checklist review form and are provided with free text boxes to elaborate on their assessment. These free text comments are reproduced below.

ARTICLE DETAILS

Title (Provisional)

Mapping methodologies for economic assessment of digital health technologies: a scoping review protocol

Authors

Santos, Ana Rita; Sampaio, Filipa; Londral, Ana; Perelman, Julian

---

VERSION 1 - REVIEW

Reviewer 1

Name Jutai, Jeffrey

Affiliation University of Ottawa Faculty of Health Sciences, Interdisciplinary School of Health Sciences

Date 07-Mar-2025

COI None

I recommend that the author consider factors that affect the adoption and diffusion of evaluation frameworks to strengthen the potential impact of the findings.

Reviewer 2

Name Solomon, Daniel

Affiliation Brigham and Women's Hospital, Division of Rheumatology, Immunology and Allergy

Date 03-Jun-2025

COI None

The authors are interested in economic evaluations of DHT, an important and interesting topic. They describe a protocol for a scoping review that proposes to review economic evaluation frameworks and their relevance to DHT.

Comments:

1. The rationale for this scoping review is not that clear. There have been other similar reviews (citations 7, 10, 11, 13). The authors mention these but do not explain the limitations or the findings of this prior very similar work. Their section on identifying the research question is weak. Their first bullet mentions finding frameworks specifically tailored for DHTs; but what if more generic frameworks work for such evaluations?

2. The search strategy suggests that mention of DHTs will be an inclusion criteria for including prior publications in this scoping review. However, previously developed economic evaluation frameworks that do NOT mention DHTs may be very adequate for assessing DHTs. DHTs are a type of health system intervention that are not so dissimilar to other previously developed interventions. This issue concerns me and weakens the rationale for the scoping review.

3. Much of the protocol reads like a textbook on how to conduct a scoping review. On the one hand this is good, but it begs the question why such a protocol is necessary (or necessary to publish). I was not convinced that I learned very much reading this protocol.

VERSION 1 - AUTHOR RESPONSE

2. Reviewer 1: Dr. Jeffrey Jutai

- **Comment 1:**

"I recommend that the author consider factors that affect the adoption and diffusion of evaluation frameworks to strengthen the potential impact of the findings."

Response:
We thank the reviewer for this insightful suggestion. We fully agree that adoption and diffusion are key factors that influence the practical relevance, applicability, and ultimate impact of economic evaluation frameworks.
In response to this recommendation, we have revised **Research Question 3** to more explicitly reflect the role of contextual factors—such as healthcare system characteristics, geographic context, and the type of DHTs being assessed—that may shape methodological decisions, including economic evaluation methods and cost components, and influence the overall suitability of different frameworks, as found on page 6:

"How does the choice of economic evaluation methods and cost components in these frameworks vary based on healthcare system characteristics, geographic context, and the specific type of DHTs assessed?"

We have also expanded the **Discussion** section to acknowledge that frameworks—even when methodologically sound—may encounter barriers to adoption depending on factors such as regulatory alignment and local priorities. These contextual dimensions will be considered in the synthesis and interpretation of our findings, in alignment with Research Question 3. Furthermore, we highlight these considerations as important directions for future research that could build on the results of this review.
As now stated in the manuscript, on page 9:

"By mapping available frameworks, this review aims to offer methodological recommendations that can guide future economic studies and support evidence-based decisions on DHTs financing, adoption and scaling up. Importantly, the review also aims to investigate how these frameworks may differ across various country contexts, health systems and DHTs types. Recognising that methodological soundness alone does not guarantee uptake, the review will consider these contextual and system-level factors as part of the synthesis and interpretation."

Reviewer 2: Prof. Daniel Solomon

- **General Comment:**

"The authors are interested in economic evaluations of DHT, an important and interesting topic."

We thank the reviewer for recognising the relevance of our topic and for the insightful feedback, which has helped us refine the manuscript.

Comment 1.1:

"The rationale for this scoping review is not that clear. There have been other similar reviews (citations 7, 10, 11, 13). The authors mention these but do not explain the limitations or the findings of this prior very similar work."

Response:
We appreciate the reviewer's comment and agree that the rationale for our review required further clarification. We have revised the **Introduction** to better distinguish our study from previous work. While some prior reviews (e.g. citations 7, 10, 11, 13) have addressed digital health technologies (DHTs) in the context of broader health technology assessment (HTA), reimbursement, or policy evaluation, none have specifically focused on identifying and analysing economic evaluation frameworks that were explicitly developed for, or clearly adapted to, DHTs.

Our review protocol addresses this gap by systematically mapping frameworks that incorporate economic aspects in the evaluation of DHTs and by analysing their methodological features, strengths, and limitations. This contribution is especially important given the complexity and uniqueness of DHTs, which pose specific challenges to conventional evaluation approaches.

As now stated in the revised **Introduction,** on pages 4 and 5:

*"This complexity presents significant challenges for conventional economic evaluation methods, particularly in selecting appropriate comparators, study designs, and perspectives, as well as accurately measuring costs and effects (15). As such, tailored frameworks are essential to address these challenges and ensure rigorous, relevant assessments of DHTs.
To the best of our knowledge, previous reviews (1,7,11,13,18,19) have addressed DHTs from an HTA or reimbursement perspective, and none have specifically focused on identifying economic evaluation frameworks that were explicitly developed for or tailored to DHTs. Some of these reviews concentrated on broader HTA domains or implementation barriers, while others examined reimbursement or value assessment without mapping the methodological structure of economic evaluation frameworks for DHTs."*

Comment 1.2:

“Their section on identifying the research question is weak. Their first bullet mentions finding frameworks specifically tailored for DHTs; but what if more generic frameworks work for such evaluations?”

Response:
We appreciate this comment and acknowledge that some generic economic evaluation frameworks may be adaptable to digital health contexts. However, the objective of this scoping review is intentionally limited to frameworks that have been explicitly developed for, or clearly adapted to, DHTs. This defined scope allows us to focus on how such frameworks respond to the unique characteristics of DHTs—such as rapid iteration, user interactivity, and the inclusion of non-health outcomes—that are often not fully addressed in more traditional or generic models.
This focus is further supported by the fact that several countries are currently developing or adopting economic evaluation frameworks specifically tailored to DHTs. For example, in the United Kingdom, NICE has issued dedicated guidelines for the economic evaluation of digital health technologies, which implicitly highlights that generic frameworks are not fully adequate for assessing DHTs. These developments underscore the need for a clearer understanding of how DHT-specific frameworks are structured and applied, and this review aims to contribute to that effort.

To clarify this rationale, we have revised the **Introduction** and **Objectives** sections. As stated in the revised text, for example, on page 5:

“By first mapping and characterising existing frameworks that have been explicitly developed or adapted for DHTs, this scoping review aims to lay the foundation for such future developments and guide methodological refinement in this evolving field.”

Comment 2:

“The search strategy suggests that mention of DHTs will be an inclusion criteria for including prior publications in this scoping review. However, previously developed economic evaluation frameworks that do NOT mention DHTs may be very adequate for assessing DHTs. DHTs are a type of health system intervention that are not so dissimilar to other previously developed interventions. This issue concerns me and weakens the rationale for the scoping review.”

Response:
We thank the reviewer for this thoughtful concern. We recognise that some existing economic evaluation frameworks—though not specifically developed for digital health— may be applicable in this context. Nonetheless, to ensure a focused and methodologically coherent review, we have decided to include only those frameworks that explicitly reference DHTs or have been adapted for their evaluation. This deliberate scoping decision is grounded in the need to understand how economic frameworks are being tailored to address the distinct methodological challenges posed by DHTs. These include fast-paced innovation cycles, evolving evidence bases, and value dimensions beyond traditional clinical outcomes. Generic frameworks that do not account for these specificities fall outside the scope of this review. We

have clarified this in the **Introduction** and **Objectives** sections. Specifically, the revised **Objectives** section now states, on page 5:

"The primary objective of this scoping review is to map existing HTA frameworks that incorporate economic aspects, such as partial or full economic evaluations, and frameworks specifically designed for the economic evaluation of DHTs. This mapping will support a better understanding of their methodological characteristics, strengths and limitations. The findings aim to inform future methodological developments and support the creation of suitable frameworks for the economic evaluation of DHTs. "

Comment 3:

"Much of the protocol reads like a textbook on how to conduct a scoping review. On the one hand, this is good, but it begs the question why such a protocol is necessary (or necessary to publish). I was not convinced that I learned very much reading this protocol."

Response:
We thank the reviewer for this valuable feedback, which we have carefully considered. We acknowledge that some sections of the original Methods text could be streamlined to avoid unnecessary details on generic scoping review procedures. Accordingly, we have revised the **Methods** section to focus more clearly on decisions and methodological choices that are specific to this review, while reducing descriptive content on general scoping review processes. At the same time, we have maintained the level of methodological transparency expected for protocol publications. As an example, the revised **Study Design** section, on page 5, now reads:

"We propose to conduct a scoping review to identify and map existing economic evaluation methodologies for digital health technologies (DHTs). Scoping reviews are particularly suited to systematically exploring the extent, range, and nature of existing or emerging literature, especially in fields where evidence is still developing (20–22). In contrast to systematic reviews, which address specific and narrowly defined research questions, scoping reviews allow for a broader examination of available evidence (23). Given that the literature on economic evaluation frameworks for DHTs has not yet been comprehensively reviewed, a scoping review is considered the most appropriate methodological approach for this study."
